# Molecular Alterations in Ferroptosis and the Effects of Resveratrol: A Systematic Review

**DOI:** 10.1002/jbt.70384

**Published:** 2025-06-30

**Authors:** Ana Beatriz Dos Santos, Júlio Santos‐Terra, Jaqueline Vieira Carletti, Iohanna Deckmann, Carmem Gottfried

**Affiliations:** ^1^ Translational Research Group in Autism Spectrum Disorder ‐ GETTEA Universidade Federal do Rio Grande do Sul (UFRGS) Porto Alegre Brazil; ^2^ Department of Biochemistry Universidade Federal do Rio Grande do Sul (UFRGS) Porto Alegre Brazil; ^3^ National Institute of Science and Technology in Neuroimmunomodulation ‐ INCT‐NIM Rio de Janeiro Brazil; ^4^ Autism Wellbeing and Research Development ‐ AWARD ‐ Initiative BR‐UK‐CA Porto Alegre Brazil

**Keywords:** animal models, ferroptosis, redox imbalance, resveratrol

## Abstract

Ferroptosis is an iron‐dependent cell death. Different from other types of cell death, ferroptosis is hallmarked by disruptions in iron metabolism, redox imbalance, antioxidant system imbalance, and lipid peroxidation. Therefore, ferroptosis triggers mitochondrial impairment, inflammation, and affects several signaling pathways. As such, modulating ferroptosis holds a promising potential for preventing its deleterious effect. Resveratrol (RSV) is a promising polyphenolic compound that modulates ferroptosis due to its chelating properties. Here, we explore the preventive effects of RSV in the molecular alterations of ferroptosis. This review systematically summarizes insights about the properties of RSV in the hallmarks of ferroptosis in animal models, highlighting its beneficial effects in modulating iron metabolism, redox imbalance, glutathione metabolism, lipid peroxidation, mitochondrial impairment, signaling pathways, and inflammation. Importantly, we emphasize the promising effects of RSV in this context, indicating its relevance in further research.

## Graphical abstract

Ferroptosis is a cell death mechanism characterized by iron overload, accumulation of reactive oxygen species, deficits in glutathione metabolism, lipid peroxidation, and mitochondrial impairments. Resveratrol can prevent or reverse these alterations by chelating iron and upregulating antioxidant defenses.

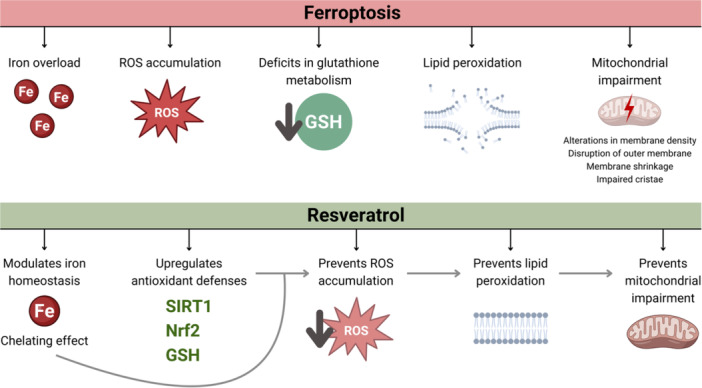

## 1 Introduction

Iron (Fe) is an essential nutrient for the human body, necessary for cellular respiration, energy production, and DNA synthesis. It also plays a role in various metabolic activities and oxidative phosphorylation, with complex storage and transport mechanisms [1, 2]. These factors make Fe an essential element for neurodevelopment. Nonetheless, both Fe deficiency and overload can be harmful. Fe levels in the human body are exclusively regulated by absorption, and studies have shown that disturbances in Fe concentration may be harmful to neurodevelopment, being associated with negative effects on neurobehavioral domains [3, 4, 5, 6, 7, 8].

Ferroptosis is a Fe‐dependent form of cell death characterized by disturbances in lipid peroxidation, increase of reactive oxygen species (ROS) accumulation, Fe overload, and impaired antioxidant defenses, mainly characterized by deficits in glutathione metabolism, with depletion of cellular Glutathione (GSH) and/or Glutathione Peroxidase 4 (GPx4) [9]. Due to disturbances in Fe metabolism, accumulated Fe levels can interact with hydrogen peroxide (H_2_O_2_), generating hydroxyl radicals via Fenton reaction, inducing tissue damage via oxidative reactions, thereby harming biomolecules [10, 11]. During ferroptosis, a Fe‐dependent lipid peroxidation is caused due to excessive ROS through the Fenton reaction, inducing cell death, which is facilitated due to a deficit in GSH antioxidant defenses [9, 12]. Studies have been suggesting the ferroptosis pivotal role in the pathology of diverse disorders, including autism spectrum disorder [13, 14, 15] and schizophrenia [16], increasingly driving the search for molecules capable of mitigating its harmful effects.

Molecules such as polyphenols, known as metal chelators, can prevent Fe accumulation and the oxidation caused by the consequent accumulation of reactive hydroxyl radicals [17]. Resveratrol (RSV) is a natural polyphenolic compound present in grapes, pines, peanuts, and red wine with antioxidant, anti‐inflammatory, and neuroprotective properties [18]. Furthermore, studies have shown that RSV reduces oxidative damage and lipid peroxidation, making it a promising molecule for ferroptosis regulation [19, 20].

In recent years, researchers have explored the beneficial effects of RSV in regulating ferroptosis in animal models, with relevant studies emerging since 2020. In this paper, we systematically review the evidence supporting the role of RSV in ferroptosis, focusing on studies that examine its effects on molecular alterations in animal models. Specifically, we: (1) analyze the main molecular pathways involved in ferroptosis in animal models, both in the absence and presence of RSV; (2) investigate the impact of RSV on key characteristics of ferroptosis, such as lipid peroxidation, Fe metabolism, oxidative stress, and deficits in GSH metabolism; (3) assess the efficacy of RSV in modulating ferroptosis across different animal models; and (4) identify gaps in the literature regarding the use of RSV in ferroptosis‐related studies in animal models.

Thus, this review aims to highlight the potential effects of RSV in regulating ferroptosis in animal models, paving the way for future studies on its protective effect against Fe‐dependent cell death.

## 2 Methodology

### 2.1 Search Strategy

This review compiles scientific studies conducted from publications inception through November 2024. It seeks to associate the effects of RSV on molecular alterations related to ferroptosis in animal models, exploring its potential applications in this context. Both search terms and selection criteria were chosen to capture all pertinent publications. The bibliographic search was carried out in the PubMed, EmBase, Scopus, Livivo, and Web of Science databases to identify publications. Both search terms and search strategies were refined to the database, developed and customized by a professional librarian, a service provided by our university. The search terms Resveratrol, Ferroptosis, and Vertebrate, along with their respective MeSH terms according to the database, were used in the search (Supporting Information S1).

### 2.2 Study Selection

The selection of studies for this review involved a rigorous process to ensure relevance and compliance with the inclusion criteria. Following removal of duplicates, the titles and abstracts of all identified publications were screened to filter out studies. This initial screening allowed for a more focused evaluation of studies that specifically examined the effects of RSV on molecular alterations associated with ferroptosis in vertebrate animal models. Subsequently, the full texts of the selected publications were reviewed for further eligibility. Both processes were independently conducted by two reviewers (DS and S‐T), blinded to each other's ratings, and minor discordances were resolved through discussion.

Inclusion criteria for the studies included: studies must (1) involve vertebrate animal models, (2) involve treatment with RSV, (3) analyze the effects of RSV on molecular alterations associated with ferroptosis in vertebrate organisms, (4) be published in peer‐reviewed journals with an impact factor ≥ 2, ensuring the scientific quality of the included publications, and (5) be available in English.

Any studies not meeting these criteria have been excluded, including: (1) studies that did not present new or unique data (such as review articles, comments, editorial, and book chapters), (2) studies that did not specifically assess the effects of RSV on molecular alterations associated with ferroptosis, (3) retracted papers, (4) studies evaluating RSV with another compound, (5) studies with invertebrate organisms, (6) studies in other language, and (7) studies that focused solely on in vitro or in silico models.

**Figure 1:**
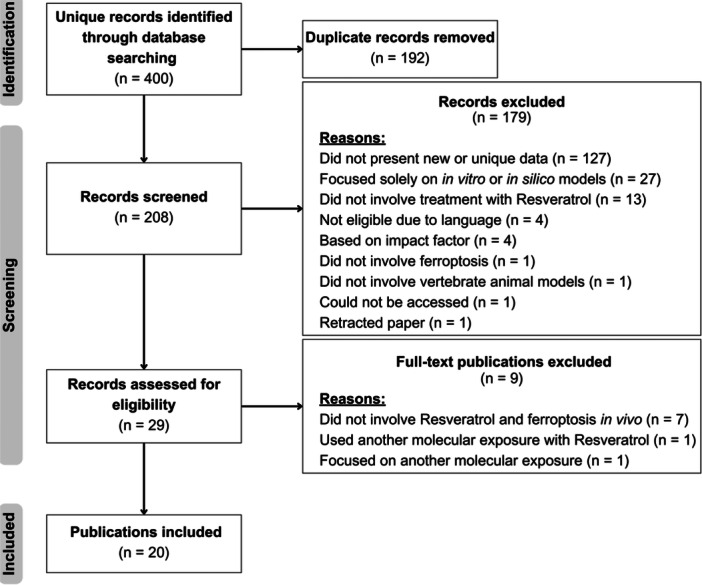
Study flowchart. PRISMA flow diagram, summarizing the identification, inclusion, and exclusion of records, along with reasons for exclusion.

A total of 20 publications met the inclusion and exclusion criteria (Figure 1).

### 2.3 Data Extraction

The included studies were fully reviewed, and the following data were extracted: author information and year of publication, type of animal model, information related to the animal models (species, age, and sex), concentration of RSV, route of administration, sample evaluated, and outcomes related to molecular alterations in the absence and presence of RSV.

## 3 Results

### 3.1 Overview of the Included Studies

Our initial search identified 400 articles, of which 20 met the eligibility criteria for inclusion in this systematic review (Figure 1). These studies utilized a variety of vertebrate animal models, with differences in RSV dosage, route of administration, and treatment duration. Specifically, 13 studies were conducted in mice, six in rats, and one in chickens. The studies were published between 2022 and 2024, reflecting growing interest in the role of RSV in ferroptosis modulation. Most studies consistently reported beneficial effects of RSV in modulating key hallmarks of ferroptosis, including disturbances in regulating Fe metabolism, ROS accumulation, GSH metabolism, and lipid peroxidation. Table 1 summarizes the main experimental parameters and outcomes across the selected studies, highlighting the diversity of pathological models, treatment protocols, and molecular endpoints. Importantly, despite differences in experimental design, a converging trend emerges: RSV exerts protective effects across a range of tissues and conditions by targeting shared ferroptosis‐related mechanisms.

**Table 1:** Comparison of ferroptosis‐related molecular alterations in the presence and absence of RSV.

Model	Animals	RSV	Administration	Sample	Molecular alterations in the absence of RSV	Molecular alterations in the presence of RSV	Author
Hypoxic‐ischemic brain injury	SD rats (7 day old) (sex 1:1)	25 µg/3 µL	Intracerebroventricularly 30 min before induction	Brain tissue	↑ SIRT1 ↑ Nrf2 ↑ Iron accumulation ↓ GPx4	↑ SIRT1 ↑ Nrf2 ↓ Iron accumulation ↑ GPx4	Li et al. 2022a
5‐fluorouracil induced cardiotoxicity	Adult male C57BL/6 J mice	1 (L), 2 (M), 4 (H) mg/kg	Intraperitoneal injection 3 weeks after induction	Plasma and heart	↑ MDA ↑ Fe^2+^ ↑ Lipid accumulation ↑ Mito impairment ↑ p53 ↑ TfR ↓ Nrf2 ↓ GPx4 ↓ GSH ↓ NQO1 ↓ FTH1	↓ MDA (M,H) ↓ Fe^2+^ (H) ↓ Lipid accumulation ↓ Mito impairment ↓ p53 ↓ TfR ↑ Nrf2 (M,H) ↑ GPx4 ↑ GSH (H) ↑ NQO1 ↓ FTH1	Li et al. 2023a
Myocardial ischemia/reperfusion	Male SD rats (10–12 week)	50 mg/kg	14 days before induction	Heart tissues	↑ MDA ↑ Fe^2+^ ↑ USP19 ↑ Beclin‐1 ↓ SOD ↓ GPx4 ↓ FTH1	↓ MDA ↓ Fe^2+^ ↓ USP19 ↓ Beclin‐1 ↑ SOD ↑ GPx4 ↑ FTH1	Li et al. 2022b
Myocardial infarction	SD rats (8 week)	20 mg/days	Injection during treatment surrounding the infarcted heart area	Heart tissue and serum	↑ IL‐6 ↑ IL‐1β ↑ MDA ↑ Lipid ROS ↑ Fe^2+^ ↓ GSH ↓ GPx4 ↓ SLC7A11	↓ IL‐6 ↓ IL‐1β ↓ MDA ↓ Lipid ROS ↓ Fe^2+^ ↑ GSH ↑ GPx4 ↑ SLC7A11	Liu et al. 2022
Endotoxemia	Male C57BL/6 mice (8–10 week)	50 mg/kg	Pretreatment with RSV	Serum and heart	↑ Relative ROS level ↑ MDA ↑ Iron accumulation ↓ GSH	↓ Relative ROS level ↓ MDA ↓ Iron accumulation ↑ GSH	Wang et al. 2022
DOX‐induced cardiotoxicity	Male C57BL/6 J mice (6 week)	30 mg/kg	Intraperitoneal injection for 3 days and then co‐treatment with DOX for 4 days	Myocardial tissue	↑ Fe^2+^ ↑ MDA ↑ Mito shrinkage ↓ GSH ↓ GPx4 ↓ p62 ↓ Nrf2 ↓ HO‐1	↓ Fe^2+^ ↓ MDA ↓ Mito shrinkage ↑ GSH ↑ GPx4 ↑ p62 ↑ Nrf2 ↑ HO‐1	Yu et al. 2022
Early brain injury after subarachnoid hemorrhage	Male C57BL/6 mice (8–10 week)	30 mg/kg	Intraperitoneal injection for 3 days before induction	Brain tissue	↑ SIRT1 ↑ MDA ↑ xCT ↓ GPx4 ↓ FSP1 ↓ CoQ10	↑ SIRT1 ↓ MDA ↓ xCT ↑ GPx4 ↑ FSP1 ↑ CoQ10	Yuan et al. 2022
Diabetes periodontitis	Male C57/BL6 mice (13 week)	100 µL (6.5 µM)	Injected locally in the periodontitis site for 7 days	Alveolar bone	↓ SLC7A11 ↓ GPx4	↑ SLC7A11 ↑ GPx4	Li et al. 2023c
Spinal cord injury	Female C57BL/6 mice (8–10 week)	200 mg/kg	Intraperitoneally injected daily for 3 consecutive days after injury	Spinal cord tissue	↑ Mito impairment ↑ MDA ↑ Fe^2+^ ↓ GPX ↓ GSH ↓ GPx4 ↓ xCT ↓ Nrf2 ↓ FPN ↓ FTH1	↓ Mito impairment ↓ MDA ↓ Fe^2+^ ↑ GPX ↑ GSH ↑ GPx4 ↑ xCT ↑ Nrf2 ↑ FPN ↑ FTH1	Ni et al. 2023
Intestinal ischemia/reperfusion	Male C57BL/6 mice (6–8 week)	30 mg/L	100 µL for 2 weeks via oral gavage	Serum and intestinal	↑ ACSL4 ↓ SIRT3 ↓ GPx4 ↓ FTH1	↓ ACSL4 ↑ SIRT3 ↑ GPx4 ↑ FTH1	Wang et al. 2023
Cecal ligation and puncture induced sepsis cardiomyopathy	Male SD rats (6–8 week)	10 (L), 30 (M), 50 (H) mg/kg	A single 2 mL dose 30 min before model induction	Heart tissue and serum	↑ Mito impairment ↑ ASCL4 ↑ Fe^2+^ ↑ MDA ↑ TFR ↓ GPx4 ↓ SIRT1 ↓ Nrf2 ↓ FTH‐1 ↓ GSH	↓ Mito impairment (H) ↓ ASCL4 (H) ↓ Fe^2+^ (M,H) ↓ MDA (M,H) ↓ TFR ↑ GPx4 (M,H) ↑ SIRT1 (H) ↑ Nrf2 (H) ↑ FTH‐1 ↑ GSH	Zeng et al. 2023
Heart failure	Male C57BL/6 J mice (2 months)	50 mg/kg	Oral gavage	Heart tissue and serum	↑ MDA ↑ IL‐1β ↑ Mito impairment ↓ SLC7A11 ↓ GPx4 ↓ GSH ↓ SOD	↓ MDA ↓ IL‐1β ↓ Mito impairment ↑ SLC7A11 ↑ GPx4 ↑ GSH ↑ SOD	Zhang et al. 2023
DOX‐induced cardiotoxicity	Male C57BL/6 J mice	20 mg/kg	Daily intraperitoneal injection 2 weeks before DOX injection	Heart tissues	↑ PTGS2 ↑ ACSL4 ↑ NCOA4 ↑ Iron accumulation ↑ ERK ↑ p38 ↑ JNK ↓ GSH ↓ GPx4	↓ PTGS2 ↓ ACSL4 ↓ NCOA4 ↓ Iron accumulation ↓ ERK ↓ p38 ↓ JNK ↑ GSH ↑ GPx4	Chen et al. 2024
T‐2 mycotoxin induced reproductive toxicity	Male C57BL/6 mice (6–8 week)	200 mg/kg	2 h before the administration of T‐2 toxin via oral gavage	Serum and testicular tissue	↑ MDA ↑ ROS fluorescence intensity ↑ Mito impairment ↑ Serum iron ↑ Fe^2+^ ↑ PTGS2 ↓ FTH1 ↓ GPx4	↓ MDA ↓ ROS fluorescence intensity ↓ Mito impairment ↓ Serum iron ↓ Fe^2+^ ↓ PTGS2 ↑ FTH1 ↑ GPx4	He et al. 2024
Myocardial ischemia/reperfusion injury	Adult male C57BL/6 mice	50 mg/kg	Intragastric injection for 14 days before induction	Serum and cardiac tissues	↑ MDA ↑ Total iron ↑ Iron contents	↓ MDA ↓ Total iron ↓ Iron contents	Hu et al. 2024
Inorganic‐arsenic induced brain injury	Female hy‐line variety white chicken (19 days)	1000 mg/kg	Mixed with chicken feed for 6 weeks during induction	Brain tissue	↑ MDA ↑ IL‐1β ↑ NF‐kB ↑ Mito impairment ↑ Iron ion ↑ DMT1 ↓ FPN1 ↓ Nrf2 ↓ SOD ↓ GSH ↓ GPx4 ↓ CAT	↓ MDA ↓ IL‐1β ↓ NF‐kB ↓ Mito impairment ↓ Iron ion ↓ DMT1 ↑ FPN1 ↑ Nrf2 ↑ SOD ↑ GSH ↑ GPx4 ↑ CAT	Pang et al. 2024
Diabetes high‐fat/high‐sugar	Male SD rats (14 week)	10 mg/kg	Daily oral gavage administration for 12 weeks before induction	Retinal tissue	↑ Mito impairment ↑ Fe^2+^ ↑ MDA ↑ PTGS2 ↓ Nrf2 ↓ GSH ↓ GPx4	↓ Mito impairment ↓ Fe^2+^ ↓ MDA ↓ PTGS2 ↑ Nrf2 ↑ GSH ↑ GPx4	Wang et al. 2024
Skin injury type 2 diabetes	Male Specific Pathogen‐Free SD rats (6–8 week)	100 μL/20 μM solution	Injected into the subcutaneous tissue around the wound every 3 days	Wound tissue (back vertebrae skin)	↑ ACSL4 ↑ MDA ↓ Nrf2 ↓ SLC7A11 ↓ GSH ↓ GPx4	↓ ACSL4 ↓ MDA ↑ Nrf2 ↑ SLC7A11 ↑ GSH ↑ GPx4	Xiao et al. 2024
Acute liver failure	Male C57BL/6 mice (8–10 week)	30 mg/kg	Intraperitoneal injection for 14 days before induction	Serum and liver tissue	↑ ACSL4 ↑ Il‐1β ↑ TNF‐α ↑ IL‐2 ↑ IL‐6 ↑ CCL2 ↑ Ac‐p53 ↑ p53 ↓ SIRT1 ↓ SLC7A11 ↓ GPx4	↓ ACSL4 ↓ Il‐1β ↓ TNF‐α ↓ IL‐2 ↓ IL‐6 ↓ CCL2 ↓ Ac‐p53 = p53 ↑ SIRT1 ↑ SLC7A11 ↑ GPx4	Zhou et al. 2024
Endometriosis	Female BALB/c nude mice (4–6 week)	25 mg/kg	Daily oral administration for 14 days	Ectopic tissues on the abdominal wall	↑ SLC7A11 ↑ GSH ↓ MDA ↓ ROS levels ↓ p53 ↓ PTGS2 ↓ Chac1	↓ SLC7A11 ↓ GSH ↑ MDA ↑ ROS levels ↑ p53 ↑ PTGS2 ↑ Chac1	Zou et al. 2024

Abbreviations: ACSL4, acyl‐CoA synthetase long‐chain family member 4; Beclin‐1, BCL2‐interacting protein 1; CAT, catalase; CCL2, C‐C motif chemokine ligand 2; Chac1, CHAC glutathione specific gamma‐glutamylcyclotransferase 1; CoQ10, coenzyme Q10; DMT1, divalent metal transporter‐1; ERK, extracellular signal‐regulated kinase; Fe^2+^, ferrous iron; FPN, ferroportin; FTH1, ferritin heavy polypeptide 1; GPx4, glutathione peroxidase 4; GPX, glutathione peroxidase; GSH, glutathione; HO‐1, heme oxygenase‐1; IL‐1β, interleukin‐1β; IL‐2, interleukin‐2; IL‐6, interleukin‐6; JNK, c‐Jun N‐terminal kinase; MDA, malondialdehyde; Mito impairment, mitochondrial impairment; Mito shrinkage, mitochondrial shrinkage; NCOA4, nuclear receptor coactivator 4; NF‐kB, nuclear factor kappa‐light‐chain‐enhancer of activated B cells; NQO1, NAD(P)H quinone oxidoreductase 1; Nrf2, nuclear factor erythroid 2‐related factor 2; PTGS2, prostaglandin‐endoperoxide synthase 2; ROS, reactive oxygen species; SIRT1, sirtuin 1; SIRT3, sirtuin 3; SLC7A11/xCT, solute carrier family 7 member 11; SD, Sprague‐Dawley; SOD, superoxide dismutase; TfR, transferrin receptor; TNF‐α, tumor necrosis factor‐alpha; USP19, ubiquitin‐specific peptidase 19.

## 4 Molecular Alterations of Ferroptosis

### 4.1 Imbalance in Iron Metabolism

**Figure 2:**
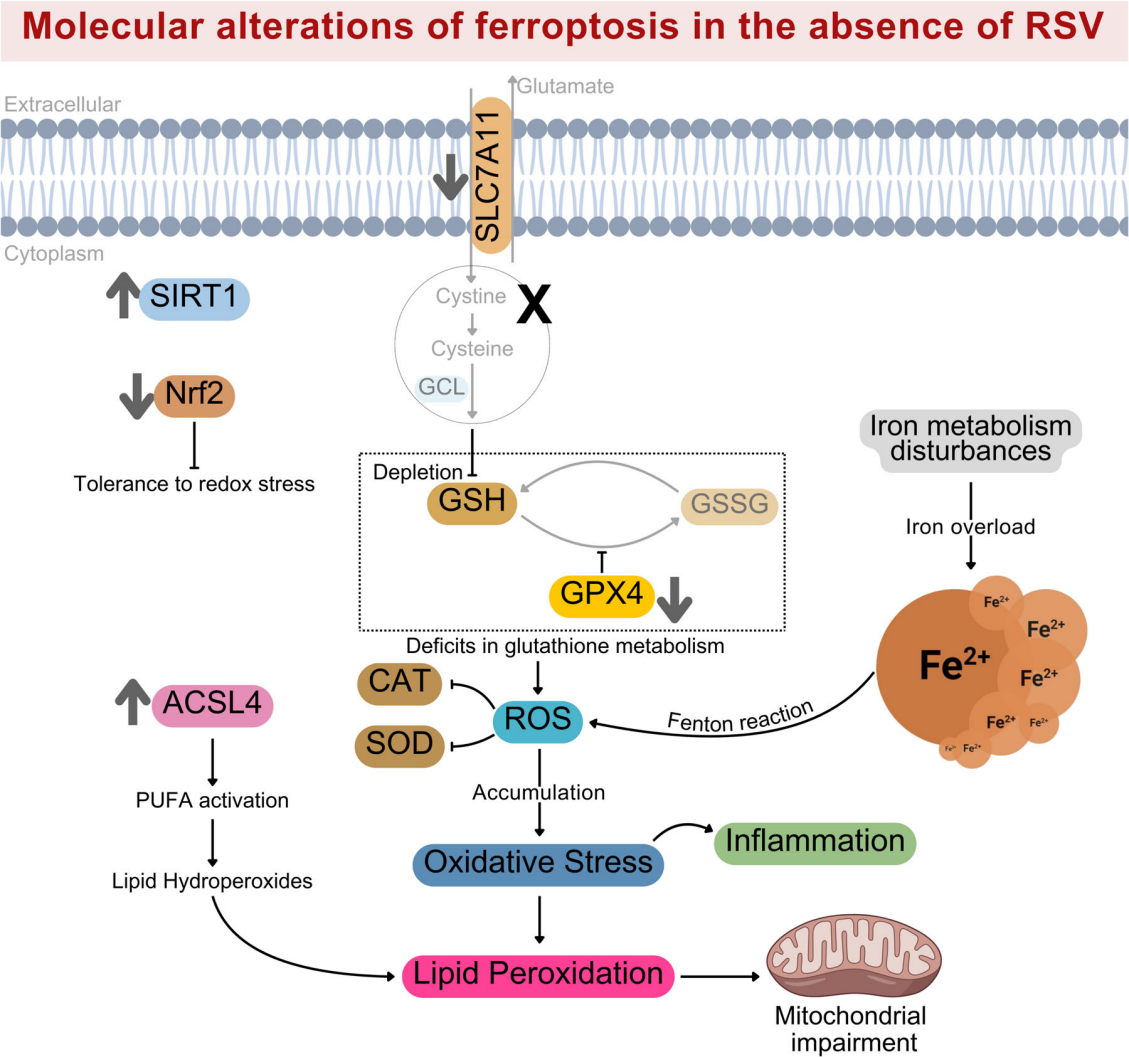
Molecular alterations of ferroptosis in the absence of RSV. Diagram of pathways relevant to the molecular alterations discussed in this review. Ferroptosis is an oxidative cell death characterized by increased ROS production, GPx4 and GSH deficits, lipid peroxidation and Fe overload. In this scenario, disturbances in iron metabolism lead to intracellular accumulation of ferrous iron (Fe²⁺), which reacts with hydrogen peroxide through the Fenton reaction, generating hydroxyl radicals that initiate lipid peroxidation. At the same time, deficits in antioxidant defenses (e.g., catalase and SOD) and GSH metabolism exacerbate ROS accumulation, further promoting oxidative stress, inflammation, and cellular damage. Cystine uptake via the SLC7A11 transporter is impaired, leading to reduced cysteine availability for GSH synthesis by glutamate–cysteine ligase (GCL). The resulting GSH depletion inhibits GPx4 activity, preventing detoxification of lipid hydroperoxides and contributing to lipid ROS accumulation. Elevated expression of ACSL4 (acyl‐CoA synthetase long‐chain family member 4) promotes incorporation of polyunsaturated fatty acids (PUFA) into membrane phospholipids, enhancing lipid peroxidation and mitochondrial damage. Additionally, downregulation of SIRT1 affects the expression of Nrf2, a transcription factor involved in redox homeostasis. Suppressed Nrf2 activity impairs the expression of target genes such as SLC7A11 and GCL, thus compounding deficits in iron, lipid, and glutathione metabolism. Collectively, these alterations converge to induce ferroptotic cell death. Abbreviations: ACSL4, acyl‐CoA synthetase long‐chain family; CAT, catalase; Fe^2+^, ferrous iron; GCL, glutamate cysteine ligase; GPx4, glutathione peroxidase 4; GSH, reduced glutathione; GSSG, oxidized glutathione; Nrf2, nuclear factor erythroid 2‐related factor 2; ROS, reactive oxygen species; SIRT1, sirtuin‐1; SLC7A11, light chain subunit solute carrier family 7 member 11; SOD, superoxide dismutase.

Fe is an essential element for vertebrate organisms, participating in mitochondrial function, enzymatic activity, and DNA synthesis [21, 22]. However, its dysregulation, especially excessive accumulation of ferrous iron (Fe²⁺), can trigger the production of ROS. These radicals initiate lipid peroxidation exacerbated by impaired antioxidant defenses, ultimately promoting ferroptosis (Figure 2). Ferroptosis is related to various biological processes, indicating its pivotal role in health through the regulation of redox balance [9, 10, 23].

Fe metabolism is rigorously regulated to limit excess Fe and ensure its role in biological processes [24], with Fe uptake and export proteins playing pivotal roles in this regulation. Transferrin receptor (TfR), divalent metal transporter‐1 (DMT1), and ferroportin (FPN) coordinate Fe uptake and efflux, while ferritin, composed of proteins such as Ferritin Heavy Polypeptide 1 (FTH1), stores excess of Fe safely. Ferritinophagy, mediated by Nuclear Receptor Coactivator 4 (NCOA4), releases Fe from ferritin when needed. Imbalances in these proteins contribute to intracellular Fe accumulation and ferroptosis [22, 25, 26].

Due its chelating properties, RSV prevents the excessive generation of toxic hydroxyl radicals, demonstrating increasing beneficial potential in regulating molecular alterations observed in the ferroptotic scenario [27]. In some animal models, RSV treatment restores Ferritin and FTH1, stabilizing Fe levels [28, 29, 30, 31, 32]. Although some studies observed partial recovery of FTH1 levels (e.g., intestinal ischemia/reperfusion model), RSV consistently demonstrated beneficial effects on Fe metabolism [33].

As NCOA4 plays a pivotal role in modulating Fe metabolism through its involvement in ferritinophagy, it is considered a key regulator of ferroptosis [22]. In some studies, the increased expression of NCOA4, leading to enhanced ferritinophagy, can be linked to Fe accumulation. RSV administration ameliorates these parameters by partially recovering NCOA4 overexpression [30, 34], suggesting a beneficial role for RSV in modulating molecular alterations associated with ferroptosis.

Studies have unveiled the regulatory role of ferroptosis in diverse pathologies, with RSV emerging as a potential molecule in preventing Fe accumulation in various diseases, including cardiopathies and those affecting nervous system, kidneys, pancreas, and reproductive system. In different animal models, RSV treatment prevents Fe accumulation [28, 29, 30, 31, 32, 35, 36, 37, 38, 39, 40, 41].

Disturbances in Fe uptake and elimination affect its concentrations, characterized by suppression of FPN and increased expression of TfR [29, 31, 32, 38]. The increased expression of DMT1 and the inhibited FPN expression reflects an accumulation of Fe ions, reverted by RSV properties in regulating Fe homeostasis [38]. The increased expression of TfR, which mainly regulates ferric iron (Fe^3+^) transport through the cell membrane, indicates disturbances in Fe transport and storage. These disturbances are also reflected in decreased FTH1 expression and Fe^2+^ accumulation. After high‐dose RSV administration, both TfR and FTH1 expression were regulated, leading to Fe^2+^ levels stabilization [29].

Fe metabolism abnormalities lead to the accumulation of Fe ions within the cell. Through oxidative reactions, excessive Fe leads to the formation of toxic hydroxyl radicals, which accumulate due to deficits in antioxidant defenses. Treatment with RSV in different animal models mitigated these disturbances by regulating Fe homeostasis, highlighting its properties in preventing Fe overload.

### 4.2 Redox Imbalance and Antioxidant Defenses

Both enzymatic and nonenzymatic antioxidant system are pivotal for combating oxidative stress, preventing ROS accumulation, and protecting against the damage induced by Fe overload and its oxidative reactions [42]. In the ferroptotic scenario, the transcription factor nuclear factor erythroid 2‐related factor 2 (Nrf2) fails to activate antioxidant mechanisms. Consequently, it is unable to regulate various ferroptosis‐related genes, including those involved in Fe metabolism, GSH metabolism, and antioxidant defense, which favors the induction of ferroptosis [31, 32, 36, 38, 39, 40, 43, 44]. Due to its antioxidant properties, RSV has shown a regulatory role in combating ROS accumulation in ferroptosis [31, 32, 36, 38, 39, 40, 43, 44].

Heme oxygenase‐1 (HO‐1) is an enzyme involved in antioxidant defense, but also plays a role in heme degradation, catalyzing its oxidative degradation and the release of Fe^2+^, thereby mediating cellular ferritinophagy and ferroptosis [45, 46]. It also contributes with the regulation of Fe levels, since in the decrease of its levels Fe tends to accumulate [45]. Yu et al. (2022) reported reduced HO‐1 levels in the absence of RSV, probably due to Nrf2 inactivation. This condition facilitates Fe^2+^ accumulation, enhancing ferroptosis‐induced damage, further aggravated by impairments in antioxidant defenses. Notably, by activating Nrf2, RSV prevents HO‐1 reduction, preventing Fe^2+^ accumulation [47].

Superoxide Dismutase (SOD), a key antioxidant enzyme, supports cellular function and integrity by neutralizing superoxide radicals, generating oxygen and H_2_O_2_ [48]. In a ferroptotic scenario, SOD levels are decreased, indicating oxidative damage. Along with the decrease in both Ubiquitin Specific Peptidase 19 and Beclin‐1 expression, damages to the defense against oxidative imbalance are detected. These impairments are ameliorated with the administration of RSV [30], recovering SOD levels in some models [49, 50]. Catalase (CAT) is an antioxidant enzyme essential for cellular defense, regulating H_2_O_2_ levels and preventing oxidative stress. Decreased CAT and SOD levels in the ferroptosis scenario are ameliorated with RSV treatment, indicating a modulation of redox imbalance [49].

Coenzyme Q10 (CoQ10) is an antioxidant involved in cellular protection by regulating redox imbalance and contributing to ferroptosis suppression [51]. Yuan et al. (2022) reported low CoQ10 protein content in the ferroptotic scenario, suggesting a potential deficit in antioxidant defenses, reflected by GPx4 inhibition and lipid peroxidation. RSV restored CoQ10 protein levels, alongside its role in suppressing ferroptosis via the ferroptosis suppressor protein 1 (FSP1)/CoQ10 system [52].

The p53 protein plays a pivotal role in Fe‐dependent cell death by inhibiting GSH metabolism, inducing ferroptosis through ROS‐induced oxidative stress [53, 54]. Ferroptosis induces hyperactivation of p53 signaling, consequently leading to impairments in GSH metabolism. In the absence of RSV, the antioxidant enzyme NAD(P)H Quinone Oxidoreductase 1 (NQO1) presents low protein levels, linked with p53 hyperactivation and Nrf2 inactivation. With RSV treatment, both NQO1 and p53 protein content are rescued [44, 55].

RSV is involved in regulating ferroptosis, but a study using a mouse model of endometriosis reported pro‐ferroptotic effects: daily administration increased p53 expression, triggered ROS accumulation, and increased the expression of CHAC glutathione specific gamma‐glutamylcyclotransferase 1 (Chac1) [56]. Chac1 is an essential regulator of redox balance and ferroptosis, and its expression is modulated by oxidative stress. The main function of Chac1 involves the degradation of GSH: its upregulation results in GSH depletion, promoting oxidative stress by diminishing the antioxidant system. The depletion of GSH affects the cells ability to counteract ROS, triggering oxidative damage and cell death [57]. These findings contrast with the properties of RSV reported in the selected studies, suggesting its potential pro‐oxidant and pro‐ferroptotic effects in certain conditions.

Ferroptosis has been linked to the triggering of the Mitogen‐Activated Protein Kinase (MAPK) pathway, which includes three subgroups: Extracellular Signal‐Regulated Kinase (ERK), c‐Jun NH2‐Terminal Kinase (JNK), and p38 [54]. Chen et al. (2024) reported increased protein levels of MAPK‐ and RSV treatment regulated such via, restoring both protein levels to those in the control group [58].

Redox imbalance is one of the hallmarks of ferroptosis, with ROS accumulation owing to impairment in the antioxidant system. Here, the studies analyzed indicate that RSV treatment attenuated redox imbalance, improving antioxidant system defenses across diverse animal models. These findings highlight the antioxidant action of RSV, suggesting its promising role in the regulation of ferroptosis by preventing antioxidant system deficits and oxidative imbalance.

### 4.3 Deficits in Glutathione Metabolism

GSH synthesis mainly involves cysteine and glycine. The Solute Carrier Family 7 Member 11 (SLC7A11, also called xCT) is a multichannel transmembrane protein and the main constituent of system xc^‐^. Such protein mediates the transport of cystine uptake and glutamate export, playing a pivotal role in GSH biosynthesis, since cystine is reduced to cysteine. Key features of ferroptosis include deficits in GSH metabolism, characterized by GSH depletion and GPx4 inactivation, contributing to ROS accumulation and lipid peroxidation [59].

Decreases in SLC7A11 activity impairs GSH synthesis due to limited cystine availability, leading to GSH depletion. Furthermore, GPx4 remains unable to protect the cell against lipid peroxidation, since GSH is unavailable as a cofactor. With RSV, these imbalances are mitigated. SLC7A11 protein levels are enhanced through SIRT1, enabling cystine uptake. With cystine levels regulated, both GSH levels and GPx4 expression are restored, which is reflected in combating lipid peroxidation [37, 50, 52, 55, 60, 61].

Despite beneficial effects of RSV in regulating GSH metabolism, Zou et al. (2024) reported different results. As mentioned above, RSV treatment increased Chac1 expression, inducing GSH depletion, further exacerbated by the inhibition of cystine uptake and the mediation of ferroptosis through SLC7A11 inhibition [56]. These findings suggest that, in this specific context, RSV may negatively modulate GSH metabolism, exerting its effects through a pro‐ferroptotic mechanism.

Nevertheless, both GSH depletion and GPx4 inhibition reported in ferroptotic conditions are counteracted by RSV treatment [31, 32, 40, 44, 47, 49, 58]. Additionally, some studies reported only a decreased GPx4 expression in the ferroptotic scenario, indicating a possible impairment in GSH metabolism due to SLC7A11 inhibition [30, 36, 43, 62]. Still, RSV treatment prevents GSH depletion and ameliorates GPx4 expression, restoring its regulatory role in lipid peroxidation.

Altogether, the studies emphasize the fundamental role of GSH metabolism imbalances in ferroptosis, with the findings suggesting that RSV modulates SLC7A11 and, consequently, the biosynthesis of GSH, highlighting its potential as a strategy to modulate ferroptosis. While some studies did not evaluate SLC7A11 expression, the observed inhibition of GPx4 indicates a potential GSH depletion via SLC7A11 inhibition. All these findings emphasize the promising role of RSV in inhibiting ferroptosis by restoring essential protective functions.

### 4.4 Lipid Peroxidation

Lipid peroxidation, a complex process involving the oxidative degradation of polyunsaturated fatty acids (PUFA) by ROS, plays a detrimental role in ferroptosis [63]. In this process, ROS accumulation initiates a chain reaction, generating harmful products that contribute to damage in cellular structures, particularly membranes, which are associated with the onset and development of neurodegenerative disorders, cardiovascular diseases, and cancer [64].

Proteins such as acyl‐CoA synthetase long‐chain family member 4 (ACSL4) and GPx4 regulate ferroptosis through their roles in PUFA metabolism and GSH biosynthesis, respectively [65]. In ferroptosis, lipid peroxidation can be triggered by excessive Fe‐dependent peroxidation of PUFA [66]. Along with ACSL4 activation, the cellular incorporation of long‐chain PUFA with other lipids and in membranes is increased [23]. This process leads to the formation of lipid hydroperoxides and secondary byproducts such as malondialdehyde (MDA), triggering cytotoxic effects along with ferroptosis [25, 67]. Consequently, this reaction can eventually lead to membrane rupture and cell death [23].

Increased MDA levels reflect lipid peroxidation in ferroptosis, with increased ACSL4 expression indicating PUFA accumulation [36, 52]. The brain is particularly susceptible to lipid peroxidation due to its high oxygen consumption, Fe content, and abundance of PUFA in neuronal membranes [64, 68]. The oxidative damage to phospholipids in lipid peroxidation leads to their degradation, which acts as a signal for cell death. Ferroptosis amplifies the role of lipid peroxidation in the biological process of cell death [69, 70].

Yuan et al. (2022) explored the potential therapeutic role of the ferroptosis inhibitor Ferrostatin (Fer)‐1 and SIRT1 activation by RSV treatment in mitigating lipid peroxidation and neuronal injury both in vitro and in vivo. Fer‐1 was found to reduce lipid peroxidation and protect neurons in both scenarios. Notably, RSV prevented lipid peroxidation, inhibiting ferroptosis and improving neurological outcomes, while inhibition of SIRT1 exacerbated lipid peroxidation and neuronal damage both in vitro and in vivo [52, 71]. In contrast to the findings from the selected studies, Zou et al. (2023) reported RSV treatment as increasing MDA levels, pointing to a potential induction of ferroptosis under these particular conditions [71].

Through its role in PUFA elimination, RSV‐mediated regulation of GPx4 may contribute to the reduction of MDA levels in ferroptosis [28, 29, 30, 31, 35, 37, 38, 39, 40, 50, 72]. These findings reinforce the ability of RSV to combat oxidative damage, in part by mitigating lipid peroxidation through its antioxidant properties. Such effect is reinforced by RSV modulating ACSL4 expression [33, 34, 55, 61, 73], preventing detrimental phospholipid oxidation.

These studies highlight the pivotal role of lipid peroxidation in ferroptosis across different animal models, with ACSL4 serving as a key factor in Fe‐dependent cell death by facilitating PUFA incorporation into membrane phospholipids, thereby increasing susceptibility to oxidative damage in ferroptosis. The accumulation of MDA further reinforces this process, emphasizing the contribution of lipid peroxidation in triggering cytotoxic effects and, posteriorly, inducing damage in the cellular membrane. Notably, RSV has demonstrated a fundamental ability to inhibit lipid peroxidation, downregulating ACSL4 expression and reducing MDA levels, thereby preventing the Fe‐dependent cell death.

### 4.5 Mitochondrial Impairment

Mitochondria are cellular organelles pivotal in energy metabolism, involved in ATP production through oxidative phosphorylation, in addition to their crucial role in maintaining redox homeostasis [74]. However, they also have an essential role in Fe metabolism: these organelles have pathways involved in heme and Fe‐sulfur cluster production, as well as in regulating Fe uptake, storage, and efflux. The regulation of mitochondrial Fe levels is crucial, since Fe imbalance impacts mitochondrial integrity [75]. Thus, preserving mitochondrial functionality, homeostasis, and morphology is vital to regulate mitochondrial quality, protect this organelle from damage, and sustain its normal morphology, structure, and function [76]. In ferroptosis, mitochondria are vulnerable to damage, leading to dysfunction characterized by morphological alterations, including membrane shrinkage, impaired mitochondrial cristae, disruption of outer mitochondrial membrane, and increased bilayer membrane density [74, 77, 78]. These morphological alterations reflect the combined effects of Fe overload, lipid peroxidation, and ROS production within the mitochondria. Taken together, the evidence highlights the significance of mitochondria in ferroptosis.

As a major contributor to ROS generation, mitochondrial metabolic alterations can trigger ferroptosis [79]. Studies in different animal models indicate that ferroptosis involves morphological and microstructure impairments in mitochondria. Li et al. (2023) reported that mitochondria were related to ferroptosis‐induced energy metabolism, observing crumpled membranes and broken mitochondria, in addition to reduced mitochondrial size and small cristae, typical characteristics of ferroptosis [29]. Otherwise, Yu et al. (2022) highlighted only mitochondrial shrinkage in the ferroptotic scenario. However, the authors call attention to other morphological alterations in ferroptosis: using H9c2 cells to evaluate ultrastructural changes in the organelle, they indicated that mitochondria and mitochondrial cristae were smaller and reduced [72].

Shrunken mitochondria are reported in ferroptotic scenarios, with reduced mitochondrial cristae and a partially ruptured outer membrane [31], increased membrane density or outer membrane rupture [32], inner mitochondrial membrane shrinkage and mitochondrial membrane rupture [50]. Accordingly, Pang et al. (2024) reported reduced mitochondrial number and volume, along with cristae rupture, mitochondria with dissolved cristae, and outer membrane rupture in the ferroptotic scenario [38]. Additionally, increased membrane density, reduced cristae, and complete cristae loss in some mitochondria were reported [40], along with diminished mitochondrial cristae, cytoplasmic and organelle swelling, and plasma membrane rupture [28]. Altogether, these studies underscore mitochondrial impairment as a hallmark of ferroptosis, potentially contributing to the subsequent cell death.

Notably, RSV treatment effectively mitigated these morphological alterations in the studies reviewed. Specifically, RSV ameliorated mitochondrial impairment, preventing mitochondrial shrinkage [28, 29, 31, 38, 50, 72, 73], cristae damage [28, 29, 31, 38, 40, 72], membrane density alterations [40, 73], and membrane rupture [28, 31, 38, 50, 73]. Preserving mitochondrial quality has become increasingly essential in protecting this organelle from ferroptosis‐induced damage, thereby maintaining bioenergetic capacity and mitigating oxidative damage [76]. Thus, the ability of RSV to activate key signaling pathways involved in Fe‐dependent cell death may contribute to preserving mitochondrial function by alleviating the hallmarks of ferroptosis.

### 4.6 Signaling Pathways

Nrf2 is associated with the cellular response to oxidative stress, displaying protective effects in several conditions, including neurodegenerative and cardiovascular disorders, diabetes, and others [80]. The main effect of Nrf2 is the increase in the transcriptional levels of genes associated with detox homeostasis, GSH synthesis, drug excretion, and heme metabolism [81]. SIRT1 is a histone deacetylase enzyme that regulates cell transcription, DNA repair, tissue regeneration, and other processes. This enzyme has been associated with neuroprotective effects [82], cardiovascular health [83], besides being involved in aging pathways [84]. Both of these molecules are involved in the ferroptosis process in different contexts.

RSV is a known activator of SIRT, which is a possible pathway by which RSV exerts its protective effects. It was already observed that RSV increases the expression of SIRT1, Nrf2, and GPx4, besides reducing Fe content in the hippocampi, indicating a protective property associated with attenuation of ferroptosis in this area. Along with that, animals treated with RSV also present improvements related to brain damage and memory/learning impairments [36]. The activation of SIRT by RSV is able to increase GPx4 and FSP1, indicating a protection against ferroptosis [52]. In both of these articles, the parallels between the treatments with RSV and Fer‐1 indicate that the modulation of the transcription induced by SIRT, activating Nrf2 and resulting (among other outcomes) in the increase of GPx4 expression is indeed a key pathway to block the progression of ferroptosis.

The role of the Nrf2‐GPx4 pathway in ferroptosis was also analyzed by Ni et al. (2023). The treatment with RSV was able to recover motor function, attenuate neuronal loss, and increase the levels of Nrf2, xCT, and GPx4. Interestingly, concomitant use of ML350, a Nrf2 inhibitor, with RSV blocked the increase in the expression of Nrf2, GPx4, and xCT, indicating the relevance of this pathway in RSV ferroptosis‐protection mechanism [31]. By modulating the Nrf2‐GPx4 pathway, RSV inhibits ferroptosis [29, 38, 40]. These improvements are not observed when a knockout for Nrf2 is used, highlighting the relevance of this transcription factor in the protective pathway [29, 38, 40].

The SIRT1‐Nrf2 signaling pathway may be important in several biological processes. RSV can regulate such a via in ferroptosis, enhancing the antioxidant defense network. When the selective inhibitor of SIRT1 EX527 is used, RSV is not able to exert its protective actions, empathizing the role of this biological pathway [32].

Overall, we can observe in several contexts that the activation of the SIRT/Nrf2/GPx4 system is the main signaling pathway by which RSV attenuates ferroptosis. Besides that, by reducing this damage, RSV also attenuates inflammation, lipid peroxidation, mitochondrial impairment, and several other deleterious effects of ferroptosis.

### 4.7 Inflammation in Ferroptosis

ROS accumulation is frequently associated with inflammation. Inflammation is a complex biological response to harmful stimuli that is essential for tissue repair and pathogen elimination, mediated by cytokines, chemokines, and transcription factors. Under physiological conditions, cytokines such as IL‐6, TNF‐α, and IFN‐γ play critical roles in activating immune cells; on the other hand, transcription factors like NF‐κB regulate the expression of pro‐inflammatory genes, modulating the intensity of the inflammatory response [85, 86].

Numerous inflammatory and degenerative conditions can be impacted by ferroptosis, due to its ability to activate the immune system, triggering a range of inflammatory factors [87]. The activation of ferroptosis can induce a pro‐inflammatory environment by promoting tissue damage and exacerbating inflammation [88], and, during these processes, ROS and oxidized lipids act as danger signals, triggering immune pathways [89]. For example, in cardiovascular diseases, Fe metabolism dysregulation exacerbates inflammation and cellular injury, furthering disease progression in conditions like myocardial infarction and heart failure [90].

Ferroptosis has been shown to contribute to inflammation by elevating the expression of prostaglandin‐endoperoxide synthase 2 (PTGS2), which encodes cyclooxygenase‐2 (COX‐2), a critical enzyme in prostaglandin synthesis. The increased expression of PTGS2 and the subsequent secretion of prostaglandin E2 accelerate arachidonic acid metabolism, leading to the release of inflammatory mediators and the activation of the inflammatory response. COX‐2, which is typically expressed at low levels under normal conditions, is rapidly upregulated in response to inflammatory stimuli such as TNF‐α and IL‐1β. Moreover, ferroptosis appears to amplify the inflammatory process through the production of lipoxygenase (LOX) and cyclooxygenase (COX) derivatives, further contributing to inflammation by increasing vascular permeability and pain sensitivity [91].

Furthermore, ferroptosis‐related treatments targeting key molecules like Nrf2 show potential for controlling oxidative stress and inflammation in various inflammatory diseases such as psoriasis, atherosclerosis, and rheumatoid arthritis [92]. Besides Nrf2, SIRT, NAD + ‐dependent deacetylases, modulate metabolic and inflammatory pathways, enhancing cellular resilience to stress [93]. Since Nrf2 and SIRT are central regulators of oxidative stress and inflammation, ferroptosis can inhibit these pathways, exacerbating inflammation and oxidative damage. In this scenario, RSV has garnered attention for its anti‐inflammatory effects across various organ systems since RSV can regulate both SIRT1 and Nrf2, suppressing ferroptosis by modulating these and other signaling pathways [94, 95].

As reviewed above, RSV can mitigate the effects of ferroptosis by diminishing the pro‐inflammatory cytokines elevating levels of IL‐1β, IL‐2, IL‐6, TNF‐α, and CCL2 [37, 38, 50, 55]. Besides, we found RSV preventive effects downregulating PTGS2 levels [28, 34, 40]. Curiously, RSV was associated with elevated levels of PTGS2 in the endometriosis model [71], with pro‐ferroptotic properties.

Through its potent antioxidant and anti‐inflammatory properties, RSV has been shown to counteract the detrimental effects of ferroptosis. By activating Nrf2, RSV promotes the expression of antioxidant genes and enhances SIRT1 activity, thereby mitigating oxidative stress and inflammation, besides protecting against ferroptosis by reducing lipid peroxidation. Additionally, by acting in PTGS2, it can modulate the COX‐2 expression, leading to a controlled response in the production of pro‐inflammatory prostaglandins. These findings highlight ferroptosis as a key mechanism in the pathology of diseases associated with Fe metabolism, oxidative stress, and immune responses while emphasizing RSV's multifaceted therapeutic potential in managing oxidative and inflammatory damage through its ability to target ferroptosis.

## 5 Effects of Resveratrol on Molecular Alterations of Ferroptosis

**Figure 3:**
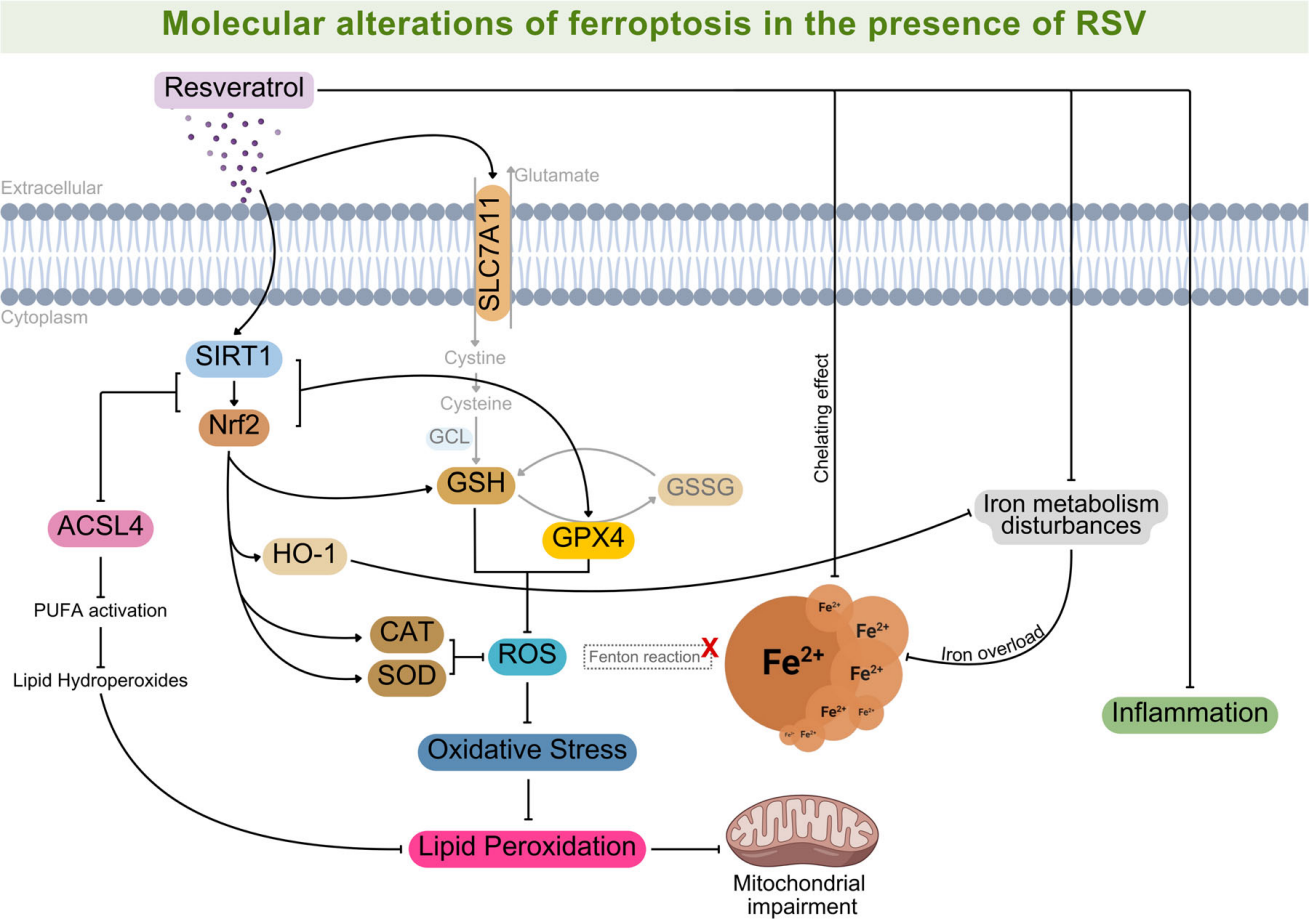
Molecular alterations of ferroptosis in the presence of RSV. RSV is a potent antioxidant and anti‐inflammatory compound, with metal chelating activity. It regulates iron metabolism by modulating proteins involved in iron uptake and export, thus preventing iron overload, and the resulting ROS generation through Fenton reaction. RSV activates SIRT1, promoting antioxidant gene expression through the Nrf2 pathway and regulating SLC7A11 expression, which restores glutathione metabolism. RSV also suppresses ACSL4 via SIRT1/Nrf2 activation, preventing PUFA oxidation, lipid peroxidation, and mitochondrial damage. Abbreviations: ACSL4, acyl‐CoA synthetase long‐chain family; CAT, catalase; Fe^2+^, ferrous iron; GCL, glutamate cysteine ligase; GPx4, glutathione peroxidase 4; GSH, reduced glutathione; GSSG, oxidized glutathione; HO‐1, heme oxygenase‐1; Nrf2, nuclear factor erythroid 2‐related factor 2; ROS, reactive oxygen species; SIRT1, sirtuin‐1; SLC7A11, light chain subunit solute carrier family 7 member 11; SOD, superoxide dismutase.

RSV is a natural polyphenolic compound with SIRT1‐activating properties [96]. This molecule has several promising therapeutic properties, with multiple health benefits through its antioxidant, anti‐inflammatory, and neuroprotective effects [18]. RSV is reported to modulate various cellular signaling pathways, activating genes involved in regulating antioxidant cascade mechanisms, such as Nrf2 signaling [97], which enhances the expression of genes associated with redox balance, glutathione biosynthesis, and Fe metabolism [81]. In ferroptosis, RSV prevents the deleterious hallmarks of Fe‐dependent cell death through its protective anti‐ferroptotic properties (Figure 3).

The antioxidant properties of RSV have been arousing interest in ferroptosis research. RSV is an efficient neutralizing agent against a range of oxidants, supporting its role as a promise agent to potentially mitigate the progression of ferroptosis [27]. Notably, RSV induces the expression of antioxidant systems and their substrates, preventing oxidative damage in the cell. This effect occurs due to the SIRT1 upregulation, which deacetylates Nrf2, promoting its transcriptional activity; in turn, Nrf2 enhances the transcription of GSH, SOD, CAT, and other antioxidant elements [98]. Besides that, Nrf2 activation also increases autophagy and prevents lipid peroxidation, improving cell metabolism in this context [99]. In addition, Nrf2 regulates the expression of ferroptosis‐related factors, including GSH metabolism via SLC7A11 activation [40]. By enhancing these defense mechanisms, RSV is involved in counteracting ROS accumulation, thereby preventing oxidative stress and mitigating cellular impairment [100].

Reactive Fe^2+^ generates toxic hydroxyl radicals via the Fenton reaction, which play a crucial role in the oxidative damage reported in ferroptosis [101]. Thus, ROS tends to accumulate, contributing to cell damage and, due to impairments in the cellular defenses, consequently leading to cell death. RSV has been shown to be effective as a scavenger of these toxic radicals, neutralizing ROS and mitigating redox imbalance associated with ferroptosis [102]. The ROS induced by Fe overload can be directly neutralized by RSV action (hydrogen or electron transfer) or indirectly neutralized by RSV due to its action in enhancing the transcription of antioxidant enzymes and other elements [103]. Hydrogen peroxide is directly correlated with ferroptosis [101], promoting ROS accumulation and impairment in cell viability by triggering oxidative reactions. RSV, in turn, is able to attenuate ROS, decreasing hydroxyl radicals generated via the Fenton reaction by regulating redox metabolism, modulating molecules such as SOD and CAT [103, 104]. By acting as a potent antioxidant, RSV protects the cell against Fe‐dependent cell death, highlighting its potential application in these scenarios.

In ferroptosis, the system xc^**‐**^ inactivation impairs GSH metabolism, leading to GSH depletion and GPx4 inactivation, which in turn promotes lipid peroxidation [59]. Activating SLC7A11 is pivotal for restoring redox homeostasis, preventing the deleterious effects of ferroptosis [105]. Due to its antioxidant properties, RSV increases the xc^**‐**^ system, promoting GSH synthesis by upregulating SLC7A11, which enhances GPx4 activity [50]. GPx4 plays a key role in lipid metabolism by inhibiting toxic lipoxygenases, leading to less reactive hydroxy derivatives, thereby preventing PUFA oxidation [106]. RSV prevents ACSL4‐derived PUFA esters increase, suppressing propagation of lipid peroxyl radicals [107, 108].

The anti‐inflammatory effect of RSV is related to its capacity to inhibit the production of pro‐inflammatory factors [109]. By modulating oxidative damage and key inflammatory pathways, RSV decreases inflammatory mediators that contribute to cellular damage in ferroptosis, thereby preserving cell integrity. Studies have reported that the anti‐inflammatory properties of RSV operate through diverse signaling pathways, such as the arachidonic acid, NF‐κB, and MAPK [110]. For example, the Nrf2 upregulation by RSV inhibits NF‐κB transcription, hindering this pro‐inflammatory pathway [111]. Specifically, by modulating these pathways, RSV may regulate the expression of pro‐inflammatory cytokines and enzymes, including COX‐2 and TNF‐α, which are often dysregulated in ferroptosis [112]. Interestingly, RSV can bind directly to COX‐2, inhibiting this enzyme [113]; regarding TNF‐α, the observed reduction is associated with the inhibition of NF‐κB [114]. Such an effect leads to a response to the inflammatory scenario, preventing an excessive immune activation, associated with pro‐inflammatory signaling.

Morphological and microstructure alterations of mitochondria are reported in ferroptosis, including fission and fusion disturbances, membrane impairments, and cristae damages [77]. Under these circumstances, RSV has come with a potential in modulating mitochondrial dynamics through its signaling pathways activation, preventing the ferroptotic damage. The peroxisome proliferator‐activated receptor gamma coactivator (PGC)‐1α is a coregulator of mitochondrial metabolism, acting as an essential transcriptional coactivator that maintains mitochondrial homeostasis. In ferroptosis, PGC‐1α activation can counteract mitochondrial impairment through its role in mitochondrial biogenesis and metabolism. As a downstream target of SIRT1, it is activated by RSV, enhancing mitochondrial protection [115, 116].

Through its involvement in mitochondrial fission and fusion processes, RSV helps maintain mitochondrial integrity. These dynamic processes are crucial, since mitochondrial fission and fusion affect the quality of mitochondria [117]. Therefore, maintaining a balance between these processes is crucial for preserving mitochondrial dynamics and function. Upon fission impairment, mitophagy is disrupted, leading to accumulation of damaged and dysfunctional organelles, which in turn triggers a pro‐oxidative scenario. Conversely, the loss of mitochondrial fusion has detrimental effects, such as oxidative stress [118]. By influencing mitochondrial dynamics, RSV is involved in mitochondrial integrity and quality, thereby reducing the accumulation of dysfunctional mitochondria that would otherwise exacerbate ROS accumulation and contribute to Fe‐dependent cell death.

The mechanisms underlying ferroptosis involve its regulation by signaling pathways, including the modulation of the voltage‐dependent anion channel (VDAC) pathway. This channel protein is located in the outer mitochondrial membrane, and mediates metabolite exchange between mitochondria and the cytosol, playing a crucial role in mitochondrial metabolism and energy production [119]. Increased VDAC activation in ferroptosis contributes to ROS accumulation and mitochondrial dysfunction [91]. RSV demonstrates a regulatory role in VDAC1, one of the three isoforms of VDAC proteins. Through VDAC1 downregulation, RSV stabilizes the mitochondrial membrane potential, thereby preventing ROS generation and mitochondrial impairments [35, 120]. These protective effects on mitochondrial dynamics are closely linked to the antioxidant and anti‐inflammatory properties of RSV, which collectively are involved in maintaining mitochondrial function under oxidative stress.

Polyphenolic compounds share common binding properties, primarily due to their metal chelating activities and affinity for proteins. However, chelated metal ions can lose their pro‐oxidant properties [17]. By chelating Fe^2+^ ions, which are involved in free radical formation, RSV can directly reduce the rate of Fenton reaction, thereby preventing oxidative damage caused by the accumulation of highly reactive radicals [121]. The critical role of metal chelation lies in its contribution to limiting the generation of free radicals toxic to cellular integrity. Thus, Fe^2+^ chelation can stabilize the oxidized form of metal ions [122]. Through its two hydroxyl groups, RSV may chelate Fe^2+^, forming a RSV‐ferrous complex [121], indicating RSV as a potential modulator of ferroptosis. Here, we highlight studies reporting that excessive Fe^2+^ exacerbates lipid peroxidation in ferroptosis, emphasizing the relevance of the properties of RSV in preventing ferroptotic cell death, alongside its role in regulating other defense mechanisms.

However, the effects of polyphenols largely depend on specific circumstances. Some polyphenols can either induce or inhibit ferroptosis, as observed with curcumin, which can promote [123, 124] or prevent [125, 126] ferroptotic cell death, according to the scenario. In cancer research, ferroptosis induction has been explored as a strategy to reduce the proliferation of cancer cells, offering a potential complementary approach to tumor treatment. Some studies suggests that RSV exhibits a bidirectional role in ferroptosis, preventing the progression of certain pathologies by promoting ferroptosis [56, 127] while also displaying anti‐ferroptotic properties, as we have explored in this review. Given the complexity of ferroptosis regulation and the multifaceted properties of polyphenols, a deeper understanding of how RSV modulates ferroptosis may pave the way for novel therapeutic strategies, reinforcing its potential in cellular integrity maintenance.

## 6 Limitations and Prospects

Using RSV as an anti‐ferroptotic agent seems to have a positive impact on the molecular alterations of ferroptosis, although current research is still advancing in elucidating its underlying molecular mechanisms. This polyphenolic compound modulates key signaling pathways involved in Fe metabolism, glutathione synthesis, antioxidant defenses, lipid peroxidation, mitochondrial function, and inflammation ‐ all of which are disrupted in ferroptosis. Targeting ferroptotic cell death appears promising in several conditions, including autism spectrum disorder (ASD). Studies have suggested a link between ASD and ferroptosis, driven by oxidative imbalances resembling those in ferroptosis, including oxidative stress, inflammation, deficits in GSH metabolism, and mitochondrial dysfunction [128, 129, 130]. Furthermore, differentially expressed ferroptosis‐related genes have been identified in ASD, paving the way for exploring diagnostic methods and therapeutic approaches [129]. In ASD and other disorders, RSV has shown a promising preventive effect on behavioral and molecular impairments, with evidence pointing to its role in modulating oxidative stress and inflammation [131, 132, 133]. While ferroptosis has begun to be explored in animal models of autism [134, 135], the interaction between ferroptosis and RSV in these models remains uninvestigated. Considering the suggested ferroptotic scenario in ASD, future research should explore the promising beneficial properties of RSV in modulating key parameters associated with Fe‐dependent cell death impairments. While targeting ferroptosis and using RSV as a potential therapeutic molecule seems promising, key issues must be addressed before clinical applications. Most ferroptosis studies rely on cell and animal models, and although RSV data indicate its potential properties in ferroptotic cell death, substantial preclinical trials are essential to assess its potential therapeutic impact in specific conditions. Considering the promising role of RSV in modulating ferroptosis, further studies are essential to investigate its potential in specific conditions. It is crucial to evaluate the efficacy of RSV as a treatment for ferroptosis‐related conditions, exploring its bidirectional role. Investigating different animal models is fundamental to determine whether RSV can be translated into an effective molecule for ameliorating specific disorders or inducing a pro‐ferroptotic effect.

## 7 Concluding Remarks

Our systematic review highlights the modulatory potential of RSV in regulating the molecular hallmarks of ferroptosis in diverse animal models. Acting through antioxidant, anti‐inflammatory, and Fe‐chelating mechanisms, RSV modulates key pathways including Nrf2, SIRT1, SLC7A11, and GPx4. Its ability to restore mitochondrial integrity, suppress ACSL4 expression, and reduce ROS and lipid peroxidation places it as a promising compound in the context of ferroptosis inhibition. Future investigations should focus on elucidating the precise molecular interactions of RSV in ferroptosis regulation, establishing optimal therapeutic windows, and validating these findings in human models. Understanding the contextual determinants of its effects will be key to advancing RSV as a potential therapeutic agent in ferroptosis‐related diseases.

## Conflicts of Interest

The authors declare no conflicts of interest.

## Supporting information

SupportingMaterial‐DosSantosetal2025.

## Data Availability

Data sharing not applicable to this article as no datasets were generated or analyzed during the current study.
