# Supplementary material for: Molecular Alterations in Ferroptosis and the Effects of Resveratrol: A Systematic Review
Source: J Biochem Mol Toxicol. 2025 Jun 30;39(7):e70384. doi: 10.1002/jbt.70384 (PMC12207836; doi:10.1002/jbt.70384)
Supplement: Supplementary file 1 — SupportingMaterial‐DosSantosetal2025. [file JBT-39-e70384-s001.docx]

**PubMed** - 45 results

**(Resveratrol**[mh] OR Resveratrol[tw] OR "trans-resveratrol"[tw] OR "trans Resveratrol"[tw] OR "resveratrol-3-sulfate"[tw] OR "3,4',5-trihydroxystilbene"[tw] OR "3,5,4'-trihydroxystilbene"[tw] OR "3,4',5-stilbenetriol"[tw] OR SRT501[tw] OR "SRT-501"[tw] OR "SRT 501"[tw] OR "SRT501"[tw] OR "cis-resveratrol"[tw] OR "cis resveratrol"[tw] OR "trans-resveratrol-3-O-sulfate"[tw] OR “3, 4, 5 stilbenetriol”[tw] OR “3, 4, 5 trihydroxystilbene”[tw]) AND (**Ferroptosis**[mh] OR Ferroptos*[tw] OR Oxytosis[tw] OR “ferroptotic cell death”[tw] OR “ferroptotic death”[tw]) AND (**Mice**[mh] OR Mice*[tw] OR Mus[tw] OR Mouse[tw] OR “newborn mice” OR **Rats[mh]** OR Rat[tw] OR Rats[tw] OR Rattus[tw] OR "Rattus norvegicus"[tw] OR "Laboratory Rat*"[tw] OR **Rodentia[mh]** OR Rodent*[tw] OR Capybara*[tw] OR Hydrochaeri*[tw] OR Jerboa*[tw] OR Dipodidae[tw] OR Beaver*[tw] OR “Hamster*”[tw] OR “gerbil*”[tw] OR "**Animal Experimentation**"[mh] OR "Animal Experimentation"[mh] OR "Animal Experiment*"[tw] OR "Animal Research"[tw] OR "**models, animal**"[mh] OR "Animal Model*"[tw] OR "Experimental Animal Model*"[tw] OR "Laboratory Animal Model*"[tw] OR “animal physical conditioning”[tw] OR “animal stud*”[tw] OR “animal trial*”[tw] OR **Animals**[mh] OR Animal*[tw] OR Metazoa*[tw] OR "**animal population groups"**[mh] OR "Animal Population Group*"[tw] OR **vertebrates[mh]** OR vertebrate*[tw])

**Web of science** - 41 results

**(Resveratrol** OR "trans-resveratrol" OR "trans Resveratrol" OR "resveratrol-3-sulfate" OR "3,4',5-trihydroxystilbene" OR "3,5,4'-trihydroxystilbene" OR "3,4',5-stilbenetriol" OR SRT501 OR "SRT-501" OR "SRT 501" OR "SRT501" OR "cis-resveratrol" OR "cis resveratrol" OR "trans-resveratrol-3-O-sulfate" OR “3, 4, 5 stilbenetriol” OR “3, 4, 5 trihydroxystilbene”) AND (**Ferroptosis** OR Ferroptos* OR Oxytosis OR “ferroptotic cell death” OR “ferroptotic death”) AND (**Mice*** OR Mus OR Mouse OR “newborn mice” OR **Rats** OR Rat OR Rats OR Rattus OR "Rattus norvegicus" OR "Laboratory Rat*" OR **Rodentia** OR Rodent* OR Capybara* OR Hydrochaeri* OR Jerboa* OR Dipodidae OR Beaver* OR “Hamster*” OR “gerbil*” OR "**Animal Experimentation**" OR "Animal Experimentation" OR "Animal Experiment*" OR "Animal Research" OR "**models, animal**" OR "Animal Model*" OR "Experimental Animal Model*" OR "Laboratory Animal Model*" OR “animal physical conditioning” OR “animal stud*” OR “animal trial*” OR **Anima**l* OR Metazoa* OR "**animal population group*"** OR **vertebrate***)

**Scopus** - 82 results

**(Resveratrol** OR "trans-resveratrol" OR "trans Resveratrol" OR "resveratrol-3-sulfate" OR "3,4',5-trihydroxystilbene" OR "3,5,4'-trihydroxystilbene" OR "3,4',5-stilbenetriol" OR SRT501 OR "SRT-501" OR "SRT 501" OR "SRT501" OR "cis-resveratrol" OR "cis resveratrol" OR "trans-resveratrol-3-O-sulfate" OR “3, 4, 5 stilbenetriol” OR “3, 4, 5 trihydroxystilbene”) AND (**Ferroptosis** OR Ferroptos* OR Oxytosis OR “ferroptotic cell death” OR “ferroptotic death”) AND (**Mice*** OR Mus OR Mouse OR “newborn mice” OR **Rats** OR Rat OR Rats OR Rattus OR "Rattus norvegicus" OR "Laboratory Rat*" OR **Rodentia** OR Rodent* OR Capybara* OR Hydrochaeri* OR Jerboa* OR Dipodidae OR Beaver* OR “Hamster*” OR “gerbil*” OR "**Animal Experimentation**" OR "Animal Experimentation" OR "Animal Experiment*" OR "Animal Research" OR "**models, animal**" OR "Animal Model*" OR "Experimental Animal Model*" OR "Laboratory Animal Model*" OR “animal physical conditioning” OR “animal stud*” OR “animal trial*” OR **Anima**l* OR Metazoa* OR "**animal population group*"** OR **vertebrate***)

**Embase** - 192 results

(**Resveratrol**/exp OR Resveratrol:ti,ab,kw OR 'trans resveratrol':ti,ab,kw OR 'resveratrol 3 sulfate':ti,ab,kw OR '3,4,5 trihydroxystilbene':ti,ab,kw OR '3,5,4 trihydroxystilbene':ti,ab,kw OR '3,4,5 stilbenetriol':ti,ab,kw OR SRT501:ti,ab,kw OR 'SRT 501':ti,ab,kw OR 'cis resveratrol':ti,ab,kw OR 'trans resveratrol 3 O sulfate':ti,ab,kw OR '3 4 5 stilbenetriol':ti,ab,kw OR '3 4 5 trihydroxystilbene':ti,ab,kw OR '5 (4 hydroxystyryl) benzene 1,3 diol':ti,ab,kw) AND (**Ferroptosis**/exp OR Ferroptos*:ti,ab,kw OR Oxytosis:ti,ab,kw OR 'ferroptotic cell death':ti,ab,kw OR 'ferroptotic death':ti,ab,kw) AND (**Mouse**/exp OR Mouse:ti,ab,kw OR Mice*:ti,ab,kw OR Mus:ti,ab,kw OR 'Mus genus':ti,ab,kw OR 'newborn mice':ti,ab,kw OR **Rat**/exp OR Rat:ti,ab,kw OR Rats:ti,ab,kw OR Rattus:ti,ab,kw OR 'Rattus norvegicus':ti,ab,kw OR 'Laboratory Rat':ti,ab,kw OR Rodent/exp OR Rodent*:ti,ab,kw OR Capybara*:ti,ab,kw OR Hydrochaeri*:ti,ab,kw OR Jerboa*:ti,ab,kw OR Dipodidae:ti,ab,kw OR Beaver*:ti,ab,kw OR 'hamsters':ti,ab,kw OR 'gerbils':ti,ab,kw OR 'South American rodents':ti,ab,kw OR '**Animal Experiment**'/exp OR 'Animal Experiment':ti,ab,kw OR 'Animal Research':ti,ab,kw OR 'animal physical conditioning':ti,ab,kw OR 'animal study':ti,ab,kw OR 'animal trial':ti,ab,kw OR 'experiment animal':ti,ab,kw OR 'physical conditioning animal':ti,ab,kw OR '**Animal Model**'/exp OR 'models animal':ti,ab,kw OR 'Animal Model':ti,ab,kw OR 'Experimental Animal Model':ti,ab,kw OR 'Laboratory Animal Model':ti,ab,kw OR 'animal disease model':ti,ab,kw OR 'model animal':ti,ab,kw OR **Animal**/exp OR Animal*:ti,ab,kw OR Animalia:ti,ab,kw OR Metazoa*:ti,ab,kw OR 'animal population group*':ti,ab,kw OR **vertebrate**/exp OR vertebrate*:ti,ab,kw)

**Livivo** - 40 results

**(Resveratrol** OR "trans-resveratrol" OR "trans Resveratrol" OR "resveratrol-3-sulfate" OR "3,4',5-trihydroxystilbene" OR "3,5,4'-trihydroxystilbene" OR "3,4',5-stilbenetriol" OR SRT501 OR "SRT-501" OR "SRT 501" OR "SRT501" OR "cis-resveratrol" OR "cis resveratrol" OR "trans-resveratrol-3-O-sulfate" OR “3, 4, 5 stilbenetriol” OR “3, 4, 5 trihydroxystilbene”) AND (**Ferroptosis** OR Ferroptos* OR Oxytosis OR “ferroptotic cell death” OR “ferroptotic death”) AND (**Mice*** OR Mus OR Mouse OR “newborn mice” OR **Rats** OR Rat OR Rats OR Rattus OR "Rattus norvegicus" OR "Laboratory Rat*" OR **Rodentia** OR Rodent* OR Capybara* OR Hydrochaeri* OR Jerboa* OR Dipodidae OR Beaver* OR “Hamster*” OR “gerbil*” OR "**Animal Experimentation**" OR "Animal Experimentation" OR "Animal Experiment*" OR "Animal Research" OR "**models, animal**" OR "Animal Model*" OR "Experimental Animal Model*" OR "Laboratory Animal Model*" OR “animal physical conditioning” OR “animal stud*” OR “animal trial*” OR **Anima**l* OR Metazoa* OR "**animal population group*"** OR **vertebrate***)
